# Current Variation in Joint Aspiration Practice for the Evaluation of Pediatric Septic Arthritis

**DOI:** 10.5435/JAAOSGlobal-D-20-00133

**Published:** 2020-09-01

**Authors:** K. Aaron Shaw, Ryan Sanborn, Benjamin Shore, Walter Truong, Joshua S. Murphy, CORTICES Study Group

**Affiliations:** Children's Healthcare of Atlanta, Department of Pediatric Orthopaedic Surgery, Dwight D. Eisenhower Army Medical Center, Fort Gordon, GA.

## Abstract

Pediatric septic arthritis (SA) is a condition that can be associated with significant morbidity. Although previous research has been on predictive care pathways, scrutiny of the literature continues to reveal wide differences in the patient evaluation and management. The purpose of this study was to define the differences in joint aspiration for the evaluation of pediatric SA across pediatric tertiary care institutions in the United States.

**Methods:**

Surgeons from 18 pediatric tertiary care centers across the United States were surveyed on current institutional practices regarding joint aspiration, laboratory studies, MRI usage, and treatment timing in the evaluation for SA. Responses were recorded by institution and analyzed to generate descriptive statistics.

**Results:**

Responses were received from all institutions asked to participate. Overall, joint specific practice variation exists regarding the person completing the aspiration, where the aspiration is performed, utilization of image guidance, and the utilization of anesthesia. Additional areas of variation included the method and calculation of cell count and the routine use of MRI.

**Discussion:**

Significant practice variations exist across pediatric tertiary care centers for the evaluation of pediatric SA. Using these data, future prospective studies can be used to unify institutional practices to minimize practice variation and ultimately improve the care delivery to pediatric patients presenting with SA.

Septic arthritis (SA) in childhood is a rare condition, but one with the potential for serious sequelae if not identified early and appropriately treated.^1-3^ Because earlier diagnosis and treatment has been shown to increase the rate of satisfactory outcomes,^2,4^ numerous attempts have been made to standardize the evaluation of children with concern for SA.^5-7^ However, without universally accepted management guidelines for pediatric SA, there remains significant variability in the proposed algorithms for clinical evaluation and management of musculoskeletal infections.^8,9,10,11^ Differences can range from units of measure for predictive markers to joint aspiration protocols for diagnostic imaging.^13,14,15,16,17,18,19^

Although institutional variation has been inferred from previous publications, no study has sought to objectively evaluate these variations in the evaluation of pediatric SA. Details about the areas of concordance and discordance in the assessment of pediatric SA will help guide protocol development efforts and drive future prospective studies.

The purpose of this study was to assess practice variations between pediatric tertiary care institutions during the evaluation and treatment of pediatric SA.

## Methods

This study was exempt from Institutional Review Board Review. A 16-question online survey (see Appendix 1, http://links.lww.com/JG9/A83) was designed and distributed to all members of the Children's Orthopaedic Trauma and Infection Consortium for Evidence-Based Studies (CORTICESs, www.cortices.org), a collaboration of pediatric orthopaedic surgeons dedicated to the improvement in quality, safety, and value in the management of emergent orthopaedic conditions across the United States. At the time of the survey, 18 pediatric tertiary care institutions based in the United States were represented*.* Questions were developed through a series of consensus discussions with experts in the field of pediatric orthopedics.

The survey (see Appendix 1, http://links.lww.com/JG9/A83) was designed to query individual institutional practice workflows and processes that are actively used in the evaluation and treatment of children suspected to have SA. Procedural information was asked regarding the performance of joint aspirations, including location in the hospital (emergency department [ED], operating room [OR], etc.) for performing aspirations, type of sedation, responsible provider performing the aspiration, and utilization of various imaging modalities. Respondents were able to select only one response for each question. Aspiration questions were subdivided according to anatomic location. Resource utilization questions were also asked, including usage of after-hours aspirations, MRI usage, and timing of surgical debridement. Responses from CORTICES members practicing at the same institution were reviewed for accuracy and internal consistency.

For each question, we documented the frequency and percentage of respondents selecting each of the given choices. Aspiration-specific and imaging response frequencies were summarized for each specific joint. Chi-square tests were used to assess differences in imaging modalities, locations, and persons across all joints and compared with each other separately. Spearman rank correlation analysis was used to assess any association between the timing of MRI and the timing of joint aspiration. Statistical significance was predetermined as *P* < 0.05. Statistical analyses were performed with R statistical software version 3.5.2 (Vienna, Austria).

## Results

Responses were received from 18 of the 18 representative institutions with a 100% response rate.

### Laboratory Units of Measure

Methods of calculating cell counts were equally split between manual differential (50%) and automated differential (50%), with the cell counts representing white blood cells (WBCs) in 71% of institutions and total cells in 29%. Neutrophil counts were recorded as a percentage of WBC in 69% of institutions and 31% representing a percentage of total cell count. Units used to report erythrocyte sedimentation rate, mm/hr, and platelets, 1000 cells/uL, were in unanimous agreement. Most institutions reported c-reactive protein levels in units of mg/dL (82%), with the remaining using mg/L (18%).

### Joint Aspiration

**Figure 1:**
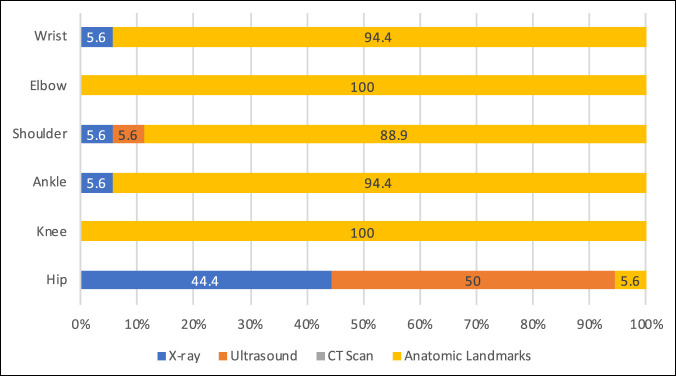
Chart demonstrating the summary of institutional responses for imaging modality used to perform joint aspirations according to specific joint of interest as a percentage of total institutions (N = 18).

**Figure 2:**
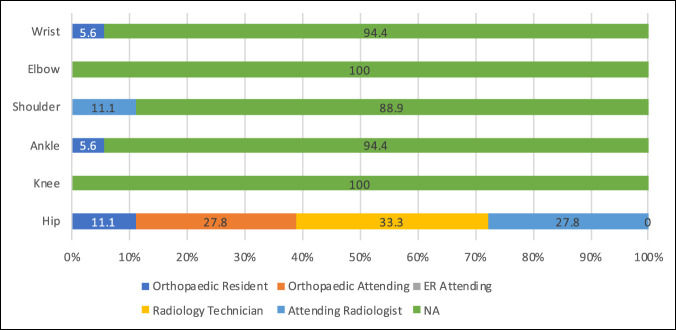
Chart demonstrating the summary of institutional responses for personnel responsible for performing imaging at the time of aspiration for the evaluation of septic arthritis according to joint of interest as a percentage of the total institutions (N = 18). ED = emergency department

Responses regarding anesthesia used for joint aspiration procedures varied according to the specified joint, Figure 1. Hip aspirations were equally performed under general anesthesia (50%) and moderate sedation (50%), whereas moderate sedation was primarily used for aspirations of the knee (55.6%), ankle (61.1%), shoulder (61.1%), elbow (55.6%), and wrist (55.6%). Image guidance usage also varied across joints with ultrasound being used for 50% of hip aspirations in comparison to knee and elbow aspirations that were performed exclusively according to anatomic landmarks, Figure 1. The provider responsible for performing imaging also varied according to the specified joint, Figure 2.

There was also significant variation in the location of aspiration procedures and responsible provider for the procedure, Table 1. Hip aspirations are performed most commonly in the OR (38.9%) by the attending staff but closely followed by interventional radiology suite (33.3%) by the radiologist and the ED (27.8%) by the resident. By contrast, knee aspirations were primarily performed in the ED (94.4%) by the orthopaedic resident (88.9%) or attending surgeon (11.1%), with no aspirations performed by the ED attending. The overall trend was for all procedures to be performed by either the orthopaedic attending, orthopaedic resident, or attending radiologist, Table 1.

**Table 1:** Summary of Institutional Responses for the Provider Who Performs Aspirations for Septic Arthritis and Where Aspirations Occur According to the Joint of Interest (N = 18)

Responses for Who Performs Aspirations for Concern of Septic Arthritis According to Joint (%)				Responses for Where Aspirations are Performed (%)		
%	Orthopaedic Resident	Orthopaedic Attending	Radiologist	ER	OR	IR Suite
Hip	27.8	38.9	33.3	27.8	38.9	33.3
Knee	88.9	11.1	0	94.4	5.6	0
Ankle	88.9	11.1	0	94.4	5.6	0
Shoulder	72.2	16.7	11.1	72.2	16.7	11.1
Elbow	83.3	16.7	0	88.9	11.1	0
Wrist	83.3	11.1	5.6	88.9	5.6	5.6

ED = Emergency Department, IR = interventional radiology, OR = operating room

### Differences Between Joints

The hip in particular showed the greatest variation in institutional practices when compared with all other joints of interest including the personnel who are responsible for performing the joint aspiration (*P* < 0.001), a technique used for joint aspiration (*P* < 0.001), and where the procedure is performed (*P* < 0.001). When assessing imaging practices, the hip again showed significant variation in institutional responses when compared against all other joints (*P* < 0.001). When assessing the remaining joints of interest, no other statistically significant differences were identified.

### Urgency of Aspirations and Formal Debridement

**Figure 3:**
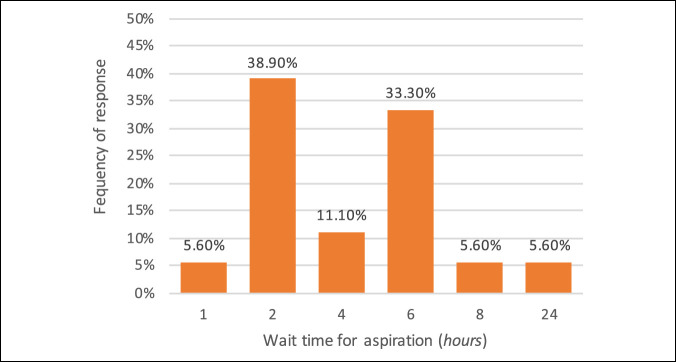
Graph demonstrating the institutional responses for the length of time, in hours, a clinically indicated patient should wait before performing an aspiration.

Institutions demonstrated varied responses about the time period when an aspiration was performed. One institution followed a protocol of performing a joint aspiration within 1 hour of suspected diagnosis and another performing within 24 hours of presentation, whereas the majority were comfortable with 2 to 6 hours, Figure 3. After hours of aspirations, defined as aspirations performed after 8 pm, for the evaluation of SA, of any joint, are performed at 94.4% of institutions. However, in hemodynamically stable patients with SA, presenting after hours, 78% of institutions indicated that they would wait until the next morning to perform a surgical debridement.

### Availability and Use of Preoperative MRI

**Figure 4:**
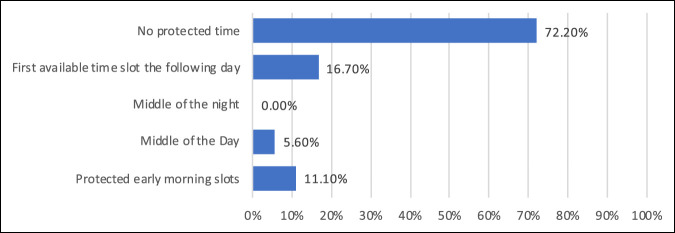
Graph demonstrating the institutional responses regarding protected MRI scheduling time slots for the evaluation of musculoskeletal infections.

When asked about MRI practices, institutions were evenly split (50%) regarding the routine use of MRI before performing either a joint aspiration or irrigation and debridement. Seventy-two percent of institutions indicated that they had no protected MRI time slots at their institutions for the evaluation of musculoskeletal infections, Figure 4. No significant correlation was noted between the availability of MRI and length of time to aspirations (R = 0.09, *P* = 0.35).

## Discussion

Through this survey, we were able to identify notable practice variability across the United States' pediatric institutions in the evaluation of children suspected to have SA. Institutional laboratory practices varied across institutions, especially for cell count. Procedural aspiration practices fluctuated according to specific joint location, with less variation existing when aspiration of superficial joints (knee, ankle, elbow, and wrist). Practice variation was the greatest when the hip joint was evaluated, especially regarding the aspiration workflows surrounding the responsible provider, imaging modality, and location for procedural aspirations. Interestingly, great range and variability was noted regarding the urgency of obtaining joint aspirations for suspected SA, but a relative consensus opinion that formal debridement of the joint could wait until the morning.

The synovial fluid WBC count is recognized as an important element of establishing a diagnosis of SA. Despite this fact, there seems to be no established pediatric threshold value for synovial WBC count to direct operative intervention in native joints.^12^ Although the traditional synovial WBC cutoff value for diagnosing SA has been set as ≥50,000 cells/mm^3^, this threshold value has not been definitively established for pediatric patients.^5^ Threshold values in the literature have been reported ranging from 50,000 to 100,000 cells/mm^3^ for diagnosing SA and values in the 25,000 to 75,000 cells/mm^3^ representing a diagnostic enigma.^12-14^ The method of performing the synovial WBC count is not universal with both manual and automated systems available. Across the 18 participating institutions, equal proportions of respondents used manual and automated synovial WBC testing. Previous studies have shown that automated cell counts have less variation in reported results while being capable of testing a larger sample size with faster performance times.^15,16^ Automated testing has also been shown to have decreased performance costs.^16^ Regarding synovial cell counts, 31% of institutions report total synovial cells instead of just WBCs, a point that could artificially inflate study results.

Previous studies have shown that the accuracy of intra-articular aspiration can vary widely depending on the joint in question and the technique used.^17,18,19,20,21,22,23^ Our results confirm wide variations in aspiration practices across different anatomic locations, with orthopaedic residents being primarily responsible for aspirations in more superficial joints (knee, ankle, elbow, and wrist) using anatomic landmarks. Previous studies have shown landmark based techniques to have an intra-articular accuracy rate for needle placement between 67% and 91% in the knee,^17-19^ 77% to 100% for the ankle,^20-22^ and 25% to 100% for the elbow.^19,22,23^ The addition of imaging guidance, be it fluoroscopy or ultrasonography, has been shown to further improve these accuracy rates.^19,24^

In comparison, the hip and shoulder aspirations showed higher rates of attending radiologist and/or surgeon participation with associated increased rates of image guidance. Leopold et al.^25^ demonstrated that landmark-based hip injections have questionable accuracy and safety. In the current study, 94.4% of institutions reported using image guidance, with 50% using ultrasound and 44.4% fluoroscopy. Ultrasound guidance has been shown in adult patients to be safe and accurate for intra-articular placement^26^ and has found growing utilization for the evaluation of the painful pediatric hip.^5,11,27^

Unlike adult patients, using local or no anesthesia is rarely sufficient in pediatric patients depending on the joint in question. As such, moderate sedation was the most commonly used anesthetic used for all joints, except the hip where a general anesthetic was most commonly used. Superficial joints with high rates of minimal or no anesthetic usage are performed almost exclusively in the ED, whereas hip aspirations are more likely to occur in either the OR or radiology suite. Phillips and Kattapuram^28^ previously showed the efficacy of hip aspirations performed by radiologists, with the benefit of lower costs and shorter hospitalization in adult patients. Kocher et al^5^ proposed a tiered, algorithmic approach for the painful, pediatric hip with recommendations for the location of aspiration, either ED or OR, based on patient symptoms, but their study did not comment on the use of interventional radiology suites. Further study comparing techniques regarding culture yields, patient satisfaction, time, and cost could guide optimal protocols.

Timing of treatment has been recognized as an important prognostic factor for final outcomes. However, a paucity of literature exists to guide when an aspiration and debridement should take place.^2,29^ This survey found varied responses in the ideal time of joint aspiration after presentation for clinically indicated patients. All but one institution reported joint aspirations were performed after hours, but 78% of institutions reported that hemodynamically stable patients with SA would await surgical debridement until the day after presentation. Previous studies have shown that articular infection produces progressive chondral destruction over time;^30^ however, other studies have shown that surgical delay of up to 48 hours in adult patients does not negatively affect outcomes.^31^

Finally, periarticular infection may be present in the setting of SA, especially in joints with metaphyseal intra-articular extension (shoulder, elbow, hip, and ankle).^32,33,34,35,36,37^ MRI has emerged as the ideal imaging modality to evaluate for adjacent infection; however, there remains some debate about the indications for, or timing of, MRI in the setting of suspected SA. Several studies have proposed guidelines for MRI acquisition,^33,37,38^ whereas other studies recommend all children with suspected SA undergo imaging because of the relative frequency of confirmed contiguous infection.^39,40^ Of the institutions queried, 50% indicated routine ordering of MRI for the evaluation of suspected infections before aspiration or surgical intervention. Furthermore, 72.2% of institutions indicated a lack of protected scheduling time for MRI. Future studies should investigate the influence of protected MRI scheduling time on the rate of acquisition of MRI, rate of identifying adjacent pathology, duration of treatment, outcomes of intervention, and cost of care.

This study is not without its limitations. As a survey, we are unable to independently confirm the generated responses. However, because many institutions are dually represented in the CORTICES membership, we were able to compare responses, increasing their accuracy. This survey was conducted to evaluate current practice experience in the evaluation and treatment of children with suspected SA. It is understood that some of the choices that are currently made by the survey respondents are because of the institutional workflows and available resources that may force the providers to practice in a specific manner. Various aspects of hospital workflow and availability of imaging, OR times are at play in the clinical decision-making process for pediatric musculoskeletal infections, including cost components and hospital efficiencies. These aspects were beyond the scope of the current study. In addition, these inquiries lacked the added benefit of how these responses might impact, if at all, patient outcomes. Furthermore, institutions were requested to provide only one answer that may represent the most common approach. These responses may not fully represent all approaches to care at each institution. Finally, the 18 institutions represented in this study, although geographically diverse within the United States, do not necessarily reflect the practice habits and workflows of other pediatric centers. These institutions represent pediatric, tertiary, academic referral centers and may not be generalizable to other types of institutions. Furthermore, all centers have access to orthopaedic surgery residents that may allow for a different approach to timing of aspirations and evaluation.

This study identified substantial workflow variation in pediatric musculoskeletal infection evaluations at large tertiary pediatric centers. From the details illuminated from this study, institutions may consider changes to decrease variability and interpretability of results, such as the use of automated cell counts and only reporting white cells in their synovial cell counts. Outlier institutions could reflect on the reasons their practice differs so greatly from others and consider a change if it is in the best interest of patient outcomes, efficiency, or cost. The broad differences in the urgency of aspirations and the utilization and timing of MRI represent areas that require further scientific investigation. The starting point in the discussion is simply to recognize the wide spectrum of practice variation, which this study has done. The next step is to engage in open dialogue as to which practices should be endorsed for widespread adoption.

In conclusion, this study identified wide variation in joint aspiration practices for children presenting to tertiary, pediatric institutions across the United States for the evaluation of SA. This information helps define the current state of practice, from which future investigations have the potential to improve efficiency and quality of care for pediatric patients with concern for SA.
